# Efficacy of smoking prevention program 'Smoke-free Kids': study protocol of a randomized controlled trial

**DOI:** 10.1186/1471-2458-9-477

**Published:** 2009-12-21

**Authors:** Marieke Hiemstra, Linda Ringlever, Roy Otten, Christine Jackson, Onno CP van Schayck, Rutger CME Engels

**Affiliations:** 1 Behavioural Science Institute, Radboud University Nijmegen, Nijmegen, P.O. Box 9104, 6500 HE Nijmegen, The Netherlands; 2 Community Health Promotion Research, RTI International, P.O. Box 12194, Research Triangle Park, NC 27709-2194, USA; 3 Department of General Practice, Maastricht University, P.O. Box 616, 6200 MD Maastricht, The Netherlands

## Abstract

**Background:**

A strong increase in smoking is noted especially among adolescents. In the Netherlands, about 5% of all 10-year olds, 25% of all 13-year olds and 62% of all 17-year olds report ever smoking. In the U.S., an intervention program called 'Smoke-free Kids' was developed to prevent children from smoking. The present study aims to assess the effects of this home-based smoking prevention program in the Netherlands.

**Methods/Design:**

A randomized controlled trial is conducted among 9 to 11-year old children of primary schools. Participants are randomly assigned to the intervention and control conditions. The intervention program consists of five printed activity modules designed to improve parenting skills specific to smoking prevention and parent-child communication regarding smoking. These modules will include additional sheets with communication tips. The modules for the control condition will include solely information on smoking and tobacco use.

Initiation of cigarette smoking (first instance of puffing on a lighted cigarette), susceptibility to cigarette smoking, smoking-related cognitions, and anti-smoking socialization will be the outcome measures. To collect the data, telephone interviews with mothers as well as with their child will be conducted at baseline. Only the children will be examined at post-intervention follow-ups (6, 12, 24, and 36 months after the baseline).

**Discussion:**

This study protocol describes the design of a randomized controlled trial that will evaluate the effectiveness of a home-based smoking prevention program. We expect that a significantly lower number of children will start smoking in the intervention condition compared to control condition as a direct result of this intervention. If the program is effective, it is applicable in daily live, which will facilitate implementation of the prevention protocol.

**Trial registration:**

Netherlands Trial Register NTR1465

## Background

A strong increase in smoking is noted especially among adolescents. Between 80,000 and 100,000 of young adolescents worldwide start smoking each day [1]. In the Netherlands, 40% of youths between the ages of 10 and 19 reports ever smoking [2]. Findings on early onset and later cigarette use suggest that those who initiate smoking in childhood are more likely to report advanced levels of smoking and nicotine dependence in late adolescence and (early) adulthood [3-6]. The consistency of findings regarding the effects of early initiation on future smoking has led investigators to advocate for a delay in the age of onset as an important strategy for preventing tobacco use.

One potential powerful tool to lower the prevalence of youth smoking and to delay the age of onset is the implementation of effective prevention programs. In the past decade, various prevention programs have been implemented primarily at secondary schools (e.g., [7]). Programs targeting on early adolescents need to be improved to be more effective [8,9]. One of the reasons that current school-based prevention programs have had little sustained effect on smoking rates is - in our opinion - the general disregard of the role of parents in preventing youth smoking onset.

Recent studies have shown that parental smoking [10], general parenting style, and parental anti-smoking socialization (e.g., [11-14]) predict smoking experimentation, progression to advanced stages of smoking, and even smoking cessation [15]. In the last five years, prospective studies have extensively studied the influence of parents on child smoking in the Netherlands. These studies generally show that parents are the primary socializing agents. Parents affect the norms of children with respect to smoking by communicating constructively about smoking-related issues, setting household rules against smoking, acquiring additional smoking-related knowledge, and monitoring their children's activities. In turn, this lowers the odds of children experimenting with smoking [11,13,16-18]. In addition to the direct influence of parents on adolescent smoking initiation, parents can also influence their children indirectly through cognitions. Anti-smoking specific parenting practices have been found to be related to adolescents' smoking-specific cognitions (i.e., social norm, self-efficacy, and attitudes [13,19], and these smoking-specific cognitions have been found to mediate the relation between parental smoking and initiation of smoking [11,17]. Considering these findings, we expect that smoking-specific cognitions will mediate the association between parenting practices and smoking initiation.

There is overwhelming empirical evidence that parents can prevent their children from smoking by engaging in anti-smoking socialization. However, no effective prevention program for parents of children aged 9-11 years old has been tested and implemented in the Netherlands. In the U.S., Jackson and Dickinson [20,21] have developed a highly innovative and successful prevention program for smoking parents of primary school children named 'Smoke-free Kids.' Smoke-free Kids is a structured program focused on anti-smoking socialization that can be conducted at home, which means that parents and children can go through these activities in their own time. Using communication, rule setting, monitoring, guided experience, and other methods of child socialization, parents can influence children's perceptions regarding the prevalence of smoking, the acceptability of smoking, and the personal and social consequences of smoking [22].

A randomized control trial conducted over a period of 24 and 36 months has provided strong evidence for the preventive effects of the Smoke-free Kids program on child smoking initiation [21]. Specifically, analyses showed that exposure to the program reduced the likelihood of children's smoking initiation at follow-up (24-months later). While 19% of children in the control condition initiated smoking by grade 6, only 12% of children in the intervention condition had done so (OR = 2.16; 95% CI 1.39 to 3.37, p < .001).

### Asthma

Health effects of smoking initiation are more profound on adolescents with asthma compared to adolescents without asthma. People with asthma who smoke are more likely to develop lung diseases and COPD [23] over time compared to those who do not smoke. Worldwide, the prevalence of asthma varies across countries and age groups. The prevalence of asthma among children aged 7-9 years old ranges from 0% to 20.3% and among 13-14 year olds from 0.1% to 16% (ISAAC study: [24]). Our institute is one of the participating collaborating parties in the ISAAC study, the worldwide epidemiological project on the prevalence of asthma and asthmatic symptoms. According to Dutch data collected from 10,087 adolescents aged 12-14 years old, 13% of the participants reported lifetime asthma and 7% reported asthmatic symptoms in the last 12 months [25]. Although one might expect that- due to the long-term negative consequences of cigarette use - adolescents with asthma would be less likely to start smoking, the contrary seems to be true (see also [26,27]). The prevalence of regular smoking among adolescents with asthma is as high as among their non-asthmatic peers [28]. In addition, adolescents with asthma are more likely to have parents who smoke than adolescents without asthma [29]. Smoking parents are less involved in anti-smoking socialization than non-smoking parents [22]. Therefore, it is important to involve this vulnerable group in smoking prevention and to examine whether the effects of the Smoke-free Kids program are different for children with and without asthma.

### Aim and hypotheses

The primary aim of the study Smoke-free Kids is to assess the effectiveness of this prevention program among children aged 9-11 years old in the Netherlands. Both short-term (after 6 months) and long-term (12, 24, 36 months) effects of the intervention will be tested. Two hypotheses will be tested. First, in line with the U.S. findings, we expect that the program will lead to lower likelihood of children's smoking initiation. Specifically, we expect that children in the intervention condition, relative to controls, will be less likely to engage in smoking at follow-up based on the findings of Smoke-free Kids in the U.S. We will test whether the effects of the intervention program are different for children with asthma. Second, we expect that the program will lead to significant increases in anti-smoking socialization of children. Specifically, we expect that parents included in the intervention program (as compared to controls) (a) will be more engaged in constructive communication on smoking topics, (b) will have more confidence in discussing smoking matters and greater self-efficacy to prevent their children from smoking, (c) will set and keep stricter household rules against smoking and establish a non-smoking contract with their children, and (e) will be more likely to monitor children's and peers' smoking-related activities.

## Methods/Design

### Study Design

The program Smoke-free Kids is a 3-year randomized controlled trial with two arms, an intervention and a control condition, testing the effects of an intervention program consisting of five activity modules. Participants consist of 1479 mothers (and their children): 729 in the intervention and 750 in the control condition. To select the eligible sample, randomization takes place at school level, to avoid contamination between conditions, after the initial recruitment and participant selection. After informed consent, enrollment in the trial, and baseline assessment, families will receive one of the five program modules every four weeks by mail. The modules for the intervention condition will consist of activities (such as games, scripted role-plays, contests, and interviews) designed to increase communication between mother and child. Mothers will be instructed to read the modules and to perform these activities together with their child. The intervention condition will also receive a booster module 12 months after the baseline to reinforce the skills learned from the earlier modules. Families in the control condition will receive modules containing only of factual information about smoking. Hence, these mothers will not be explicitly encouraged to communicate about the modules with their child.

**Figure 1:**
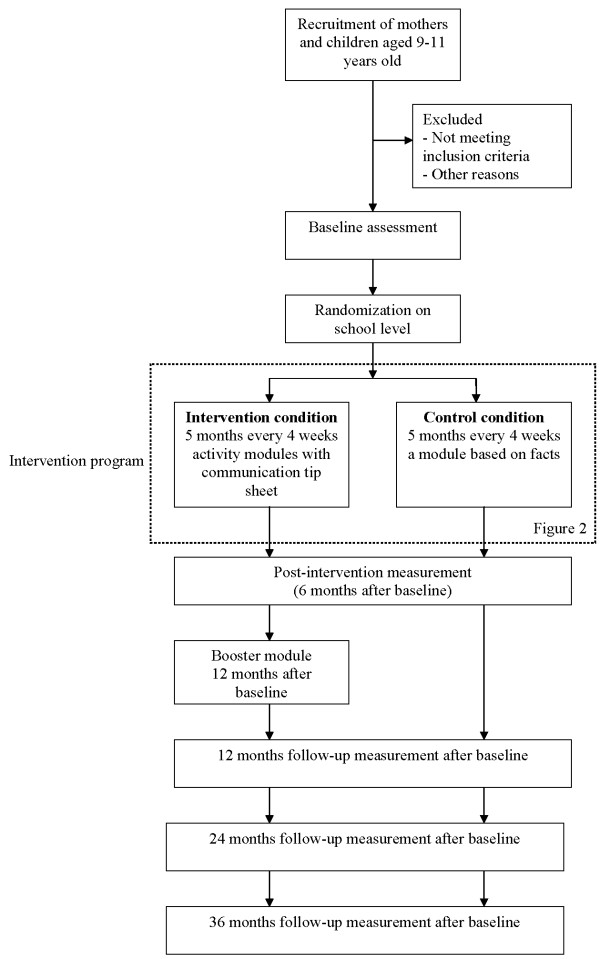
**Study design**.

Assessments in both intervention and control condition will be conducted at baseline, after six months (after completing the intervention), 12, 24, and 36 months (see Figure 1).

After 36 months of follow-up, each family will receive €10 for participation in all measurements, and five traveler's cheques of €1000 will be raffled among these families. Children will receive little gifts after different measurement (e.g., pen & memo pad, magnet stickers, Frisbees) to thank them for participating in the study.

### Participants

#### Recruitment

Families are recruited from primary schools, media, and health professionals. Specifically, primary school boards are asked to distribute letters to all children aged 9-11 years old and to request that children give this letter to their parents. This letter includes information about the study and inquires whether parents want to be involved in our study. If parents agree to participate, they can provide their contact information by filling out a short screening self-administered questionnaire (that includes items assessing parental smoking status and possible asthmatic symptoms of the child) and return it in the enclosed envelope. It is also possible to register online via a secured webpage. To recruit children with asthmatic symptoms, several local and national newspapers, a local television station, and different health related prevention websites (e.g., Dutch Asthma Foundation, Dutch Institute for Smoking Prevention) agreed to assist in announcing the study on a population level. Furthermore, health professionals (i.e., general practitioners, pharmacist, and lung specialists) are requested to place posters with accompanying flyers in their waiting rooms.

#### Eligibility Criteria

Eligibility is determined in two steps; first based on a short screening self-administered questionnaire completed by the parents, and second based on the baseline telephone interview. Inclusion criteria for the present study are; children have to be aged between 9-11 years old and should not have initiated smoking yet, participating adults have to be the mother or a female guardian, and both adult and child need to be competent in reading and speaking Dutch. Furthermore, only one child per household is eligible to participate. To test the moderating effect of asthma, we also needed a subsample (*n *= 200) of children with asthmatic symptoms. Written informed consent from participating families will be obtained upon enrollment. The ethics committee of the Faculty of Social Sciences at the Radboud University Nijmegen approved the study's protocol.

In this study, we will focus on 9 to 11 year old children because at this age, children start to become increasingly interested in smoking issues (see [30]), but generally do not smoke yet. The prevalence of lifetime smoking among this age group is low (< 2%) [31], making it an important target group for primary prevention. Furthermore, this age group consists of children prior to the phase in which they enter pre-puberty. This is a period characterized by increasing conflicts with parents, particularly with mothers [32], leading to less conformity and openness, although children are still responsive to the influence of parents [21].

We have decided to target mothers rather than fathers for the following reasons; (a) if parents are divorced, children live mostly with their mothers [33], (b) on an average, children spend more time with their mothers than with their fathers, which gives mothers the practical advantage of having more time to deliver the anti-smoking socialization program to their children, [34] (c) women are generally more likely than men to enroll in health-related programs, (d) the U.S. trial also included only mothers, so including mothers would increase comparability of findings [20,21], and (e) given the plausibility that program effects would differ by parent's gender, including fathers would substantially increase the size and costs of the proposed trial.

#### Randomization

Randomization occurs at the school level to avoid contamination between conditions. Thus, clusters of children from one school are allocated to either the intervention or the control condition. An independent statistician performed the allocation and stratified participants by school and number of children with asthma after the baseline assessment.

### Sample Size Calculation

Based on the findings from the U.S. trial, we expect a 10% difference in smoking initiation rates between the intervention and control conditions. Equal cell sizes are assumed for study cells and power of .80 was targeted. The primary hypothesis will be tested at an overall two-sided significance level of 0.05. We used the general-purpose statistical software package Stata to calculate the estimated sample sizes for two-sample comparison of proportions. Based on the U.S. data and the prevalence of smoking in 12 to 14 year olds (age of the children at 36-months follow-up), which is around 30%, we would need 428 children per condition. In these power analyses, we corrected for the fact that data are clustered (children are nested within schools) and the fact that we will apply multiple imputation in the case of missing data. Thus, 856 children (and mothers) would be included to test the effectiveness of Smoke-free Kids. A sub-goal of the study is to examine whether there is any difference between the children with and without asthma or asthmatic symptoms. To test the moderating effect of asthma or asthmatic symptoms, we will include a subsample of 200 children with asthma or asthmatic symptoms. This allows us to test whether the effect of the intervention is different for children with asthma. Eventually, the study is over-enrolled. Overall, a total of 1479 children (and mothers) will participate in the study: 1399 never smokers and 80 ever smokers. The asthmatic subsample includes approximately 239 children whose mothers reported their child to have had an asthmatic period at least once in their lives. This allows us to test whether the effect of the intervention is different for children with asthma. Moreover, having 623 additional participants allows us to do complex analyses and to test several other moderators and mediators. In accordance with the intention-to-treat philosophy, all children randomized to one of the conditions are included in analyses to test the study hypotheses.

### Study intervention

#### Theoretical basis of the intervention

Social Cognitive Theory [35] and models of persuasive communication for attitude and behavioral change [36] were used to structure the program to meet the intervention objectives. Bandura's Social Cognitive Theory (1986) has been broadly applied in public health intervention, and it has been used here to identify the critical elements of child socialization regarding cigarette smoking. Specifically, these elements include a) perception, where a child perceives the expressed thoughts and actions of parents or other socializing agents, b) cognitive rehearsal, where a child recalls and assigns meaning to what has been perceived, c) behavioral rehearsal, where a child communicates or acts in a manner consistent with what has been learned and receives feedback regarding those thoughts and behaviors, and d) motivation, where a child experiences positive (or negative) reinforcements for specific communications or actions. Each element of the program was designed to address one or more of these child socialization processes.

Communication models, particularly the Elaboration Likelihood Model [36], offer substantive input as regards the design of persuasive communications. Of particular importance is that participants vary with respect to the perceived relevance and salience of health communications, and the intervention design should take this variability into account. For example, we expect some parents to engage in argument-based processing of program content (where message content most affects parental response to program recommendations), and others to engage in cue-based processing (where peripheral cues such as print design most affect parental response to program recommendations). The program information has been structured to address both modes of information processing.

#### Intervention Condition

Parents and children in the intervention condition will receive five printed activity modules by mail at four-week intervals. The aim of the modules is to achieve progressive development of parent-child socialization activities. Activities have been designed to gradually increase parental skills and comfort level in communicating with children about smoking, addiction, and expectations regarding abstinence. Each activity module includes a high concentration of structured interactions that engages parent and child simultaneously, such as games, scripted role-plays, contests, and interviews. These structured interactions are a key technique for facilitating parent-child engagement in the intervention [37].

Each module aims to modify different socialization variables, module 1 targets general communication about smoking and makes parents and child comfortable with communicating about smoking, module 2 concentrates on influence of smoking messages (i.e., influence of media, sport events, and people around us), module 3 focuses on setting rules about smoking to protect their child from experimentation with tobacco. Module 4 is an extension of module 3 and involves creating a smoke-free house and -environment to keep the child away from second hand smoking. The last module, number 5, increases children's awareness regarding the influence of smoking classmates and friends and increases their ability to handle peer pressure. All five modules contain a communication sheet for parents. These sheets provide additional background information about the subjects discussed in the modules and communication tips for parents. Finally, a booster module will be delivered 12-months post-baseline.

#### Newsletters

Between the activity modules, parents will receive a series of digital newsletters in their email box. These newsletters will be sent after modules two, three, and five. The newsletters aim to maintain commitment to the program. The newsletters will inform parents about the background of Smoke-free Kids, review the activity modules that parents and child receive in the mail, and announce the winners of different program contests (e.g., 'drawing an anti-smoke message,' 'compose the longest sentence with magnet stickers,' and 'writing a story including an anti-smoking message').

#### Booster module

Evaluations of smoking prevention programs for adolescents indicate that repeated exposure to the key elements of the intervention program can strengthen program effects. A booster module will be developed with the theme 'Staying smoke-free.' This module includes a self-assessment component; i.e., parents and children will evaluate which anti-smoking skills they have practiced well, and which ones they could improve. Additionally, motivational information to stay smoke free throughout the high school years will be provided.

#### Control condition

**Figure 2:**
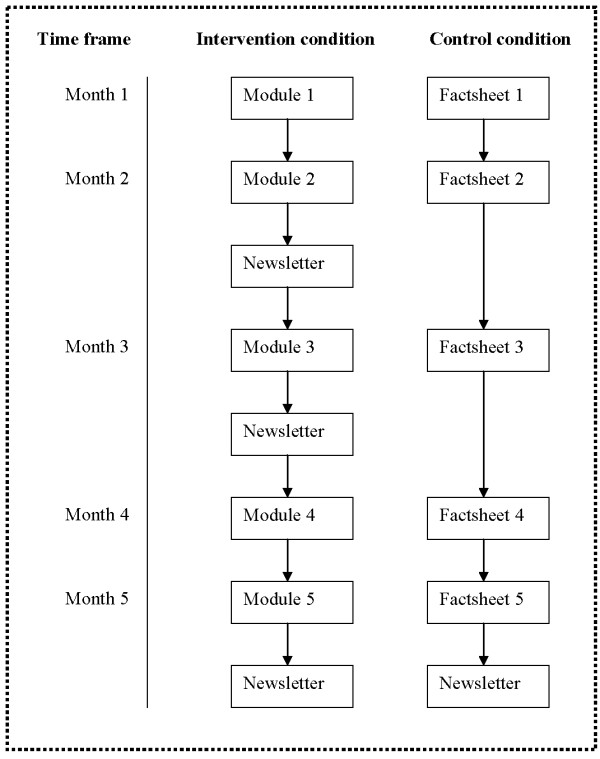
**Intervention program**. An overview and time frame of the intervention program.

For the families in the control condition, a fact-based program has been developed. An alternative program will be provided for controls because we assume it is unethical to recruit them for an intervention program while not offering them a program afterwards. Providing alternative materials for controls also helps maintain comparable response rates when follow-up data are collected from the two arms of the study. The factsheets provide information on youth smoking and focuses parents' attention on macro-level variables relevant to youth smoking, but not targeted by the intervention version of the program (for example, smoking prevalence among youths, ingredients of cigarettes, tobacco legislation). The criterion for selecting factsheets information was that the same information would be available in local, state, or national print or broadcast media. Although the information provided could increase control condition parents' knowledge regarding tobacco issues, this awareness is not expected to affect anti-smoking socialization processes. Moreover, it is difficult to retain parents in the study without providing them anything of a program. Both factsheets and modules will be mailed at the same time to participants in the control and intervention condition (Figure 2). Similar to the children in the intervention condition, the children in the control condition will also receive incentives (magnet stickers & Frisbees) to thank them for participating.

All the U.S. materials were translated and adapted to the Dutch language. This was done in collaboration with STIVORO (Dutch Institute for Smoking Prevention), the Trimbos Institute (Netherlands Institute of Mental Health and Addiction), and professional translators. The following adaptations were made for the Dutch intervention. For instance, some assignments were not suitable for the Dutch intervention because they were too culturally specific or they concerned issues that have changed since the U.S. program started. For instance, the U.S. intervention included assignments that referred to tobacco advertising, which is prohibited nowadays. Moreover, while the original program targeted smoking mothers, the Dutch program was made accessible to both smoking and non-smoking mothers; therefore, the focus of some modules needed to be changed. Finally, the layout of the modules has been modernized and adapted (i.e., cartoons).

### Data collection

An overview of all measurements is given in Table 1. All questions will be administered during a 20-minutes telephone interview by one of the trained interviewers. At baseline, mothers will be interviewed first to check the eligibility of the family. Children will be interviewed few days later. Because of practical reasons, the over-enrolled families will be asked to answer the questions by questionnaire which will be sent to their homes. Only the children will be examined at post-intervention and follow-ups. We considered collecting data from parents at each follow-up, but we opted not to because (a) such data are not needed to test the study hypotheses and (b) our perspective is that children's perceptions of anti-smoking socialization are more reliable (less biased) and will explain their smoking status better than parental reports of anti-smoking socialization (see also [38-40]).

**Table 1:** Overview of measurements

Measurement	Baseline		Post-intervention (6 months after baseline)	Follow-up I (12 months after baseline)	Follow-up II (24 months after baseline)	Follow-up III (36 months after baseline)
	Mother	Child	Child	Child	Child	Child
Demographic characteristics	X					
Smoking behavior parents [57]	X	X	X	X	X	X
Smoking behavior child [57]	X	X	X	X	X	X
Anti smoking socialization:						
Communication about smoking [46]	X	X	X	X	X	X
Monitoring [19]	X		X	X	X	X
Availability of cigarettes at home [19]	X	X	X	X	X	X
Parental norms [19]	X		X	X	X	X
Parental influence on their offspring smoking [19]	X	X	X	X	X	X
House rules [19]	X	X	X	X	X	X
Constructive reaction: perceptions of the parents reaction [19]		X	X	X	X	X
Intention to smoke [58]		X	X	X	X	X
Self-efficacy [49,59]		X	X	X	X	X
Attitude [60]		X	X	X	X	X
Social Norm [61]		X	X	X	X	X
General Parenting Style	X	X	X	X	X	X
Smoking behavior peers [62]		X	X	X	X	X
Parent-child relationship (NRI) [63]	X	X	X	X	X	X
Alcohol use		X		X	X	X
Strength and Difficulty Questionnaire (SDQ) [51]	X	X		X	X	X
Asthmatic symptoms (ISAAC) [50]	X	X		X	X	X
Program Evaluation/utilization [20]			X			

During the intervention program, 10% of the participants in the intervention condition will receive a telephone call from a trained interviewer about the procedure of the program. They will be asked if they received the activity modules and which modules they did utilize so far. The answers will give us an indication about program exposure among intervention condition families.

The post-intervention measurement (after 6 months) will collect more detailed information on program utilization. The three follow-up measurements will be at 12, 24, and 36 months after baseline. We have decided to follow the children for 36 months, indicating that at the final wave, children will be 12 to 14 years old. The national prevalence data on smoking in adolescents have shown an increase in ever smoking of 5% among 10-year olds, 7% among 11-year olds, 17% among 12-year olds, and of 25% among 13-year olds [2].

### Outcomes

The primary outcome, initiation of cigarette smoking, has been defined as puffing on a lighted cigarette for the first time. Secondary outcome measures are general parenting dimensions like monitoring, psychological control, manipulative control, support and responsiveness (e.g., [41-44]), as well as smoking-specific parenting, such as house rules on smoking, non-smoking agreement, warnings about consequences of smoking, frequency and quality of communication on smoking matters, and reactions on experimentation with smoking (e.g., [11,17,19,45-47]). Other outcomes are susceptibility to cigarette smoking, defined as the lack of a firm commitment against cigarette smoking [9,48], child smoking-related cognitions, such as expectancies concerning self-efficacy [49], and social norms [13] which have been shown to be related to smoking initiation [12,28] and attitude [13]. Asthma symptoms will be identified using an extended version of ISAAC's asthma questionnaire [50]. In addition, children with asthmatic symptoms will be phenotyped using lung function measurement. The Strengths and Difficulties Questionnaire (SDQ) [51] will be used as a behavioral screening instrument for early detection of psychological problems. Psychological problems are associated with problem behaviors like smoking (e.g., [52]).

### Statistical analyses

The main comparisons of study conditions with respect to the distribution of time until first instance of smoking will be based on survival analysis methods. All available data for participants who are randomized but lost to follow-up will be used in the survival analysis. This way, if a participant is not able to be located after the first year, for example, the data collected from the participant up to one year will be used in estimating the intervention effect and will contribute to the time trend estimates up to a year. Survival analysis is selected as the primary analysis in part because it easily incorporates censored observations. Logistic regression models will also be used to test how the intervention is related to susceptibility of smoking in originally abstinent children. Mplus analyses will be used to deal with missing data at the subsequent waves and to control for the clustered data (e.g., the fact that we randomize on school level) (see [53]).

### Time Frame

The recruitment, inclusion, randomization of participants started at the end of 2008. The final follow-up measurement is planned for mid-2012. All data will be continuously collected, entered, and cleaned. Short-term results will be reported before the completion of the 36 months follow-up.

## Discussion

The present study protocol presents the design of a randomized controlled trial evaluating the effectiveness of a smoking prevention program for 9 to 11 years old children. The intervention program called 'Smoke-free Kids' aims to prevent children from initiating smoking. It is hypothesized that, after three years of follow-up, children in the intervention condition will be less likely to initiate smoking, and that maternal communication about smoking topics, confidence in discussing smoking, and efficacy to prevent their children from smoking will increase compared to the control condition.

### Strengths and limitations

An important first strength of Smoke-free Kids program is that the program is theory-driven. Social Cognitive Theory [35] and models of persuasive communication for attitude and behavior change [36] have been used to structure the intervention. Second, the program is a home-based prevention program, which means that parents and children can go through the activities on their own, in their leisure time, and are not obligated to engage in a complex, time-consuming program. Third, this program focuses on children who have not initiated smoking yet.

Strength of the study design is that it includes follow-up measurements at 6, 12, 24, and 36 months, which allows us to test the short and long-term effects of the intervention program. Second, regarding the generalizability of the study results, if Smoke-free Kids is effective, the program can be easily implemented in the home setting and disseminated, for example, by primary schools, general practitioners, and school doctors. A limitation of the study is that the behavior of the children and parents is based on self-reports. However, studies have shown that self-reported data of adolescents about their own smoking are generally reliable [54-56].

### Implications for practice

If the Smoke-free Kids intervention program is effective, it could be easily applied to daily life, which will facilitate implementation of the prevention protocol. The program's modular, self-help format allows flexibility as regards where, when, and how it is implemented. Although the present study will measure effects on individual children after delivering the modules to households, in the future, the program could also be delivered to multiple families at the group-level using an alternative approach (e.g., at school), or it could be self-administered on a website that provides sequential access to the intervention modules. This is the reason that STIVORO (Dutch Institute for Smoking Prevention) and the Trimbos Institute (Netherlands Institute of Mental Health and Addiction) are actively involved. This all implies a strong potential of the program to reach large populations. In addition, if the home-based prevention program is effective, it can be developed for other risk taking behavior like alcohol and drugs.

## Conclusion

This study will evaluate a protocol for preventing smoking initiation in children. The results of this study will provide insights into the effectiveness of the Smoke-free Kids intervention program and the precursors of smoking initiation among children aged 9 to 11 year olds.

## Competing interests

The authors declare that they have no competing interests.

## Authors' contributions

MH and LR are responsible for the data collection, data analysis and for reporting the study results. CJ is the principal investigator of the Smoke-free Kids trials in the U.S. RO, OS, and RE are supervisors and grant applicators. All authors read and approved the final manuscript.

## Pre-publication history

The pre-publication history for this paper can be accessed here:

http://www.biomedcentral.com/1471-2458/9/477/prepub
